# Dual targeting of androgen receptor and mTORC1 by salinomycin in prostate cancer

**DOI:** 10.18632/oncotarget.11404

**Published:** 2016-08-19

**Authors:** Nooshin Mirkheshti, Sulgi Park, Shoulei Jiang, Jodie Cropper, Sherry L. Werner, Chung S. Song, Bandana Chatterjee

**Affiliations:** ^1^ Department of Molecular Medicine, University of Texas Health Science Center at San Antonio, San Antonio, Texas 78245, USA; ^2^ Department of Pathology, University of Texas Health Science Center at San Antonio, San Antonio, Texas 78245, USA; ^3^ South Texas Veterans Health Care System, San Antonio, Texas 78229, USA

**Keywords:** androgen receptor, CYP17A1, HSD3β1, mTORC1, AMPK

## Abstract

Androgen receptor (AR) and PI3K/AKT/mTORC1 are major survival signals that drive prostate cancer to a lethal disease. Reciprocal activation of these oncogenic pathways from negative cross talks contributes to low/limited success of pathway-selective inhibitors in curbing prostate cancer progression. We report that the antibiotic salinomycin, a cancer stem cell blocker, is a dual-acting AR and mTORC1 inhibitor, inhibiting PTEN-deficient castration-sensitive and castration-resistant prostate cancer in culture and xenograft tumors. AR expression, its transcriptional activity, and androgen biosynthesis regulating enzymes CYP17A1, HSD3β1 were reduced by sub-micro molar salinomycin. Estrogen receptor-α expression was unchanged. Loss of phosphorylated AR at serine-81, which is an index for nuclear AR activity, preceded total AR reduction. Rapamycin enhanced the AR protein level without altering phosphoAR-Ser81 and CYP17A1. Inactivation of mTORC1, evident from reduced phosphorylation of mTOR and downstream effectors, as well as AMPK activation led to robust autophagy induction. Apoptosis increased modestly, albeit significantly, by sub-micro molar salinomycin. Enhanced stimulatory TSC2 phosphorylation at Ser-1387 by AMPK, and reduced inhibitory TSC2 phosphorylation at Ser-939/Thr-1462 catalyzed by AKT augmented TSC2/TSC1 activity, which led to mTORC1 inhibition. AMPK-mediated raptor phosphorylation further reduced mTOR's kinase function and mTORC1 activity. Our novel finding on dual inhibition of AR and mTORC1 suggests that salinomycin is potentially active as monotherapy against advanced prostate cancer.

## INTRODUCTION

Androgen receptor (AR) and Mechanistic Target of Rapamycin Complex-1 (mTORC1) activities are two key drivers of prostate cancer. Apoptosis of castration-sensitive prostate cancer cells in response to androgen deprivation therapy (ADT) causes initial regression of the disseminated disease, although recurrence to a castration-resistant lethal phenotype is inevitable, and for ~70% post-ADT patients cancer progresses within 20 months [1, 2]. Persistent expression of AR and its restored activation by de novo synthesized tumor tissue androgens are among the major triggers of disease progression to metastatic castration resistant prostate cancer (mCRPC) [3, 4]. Inhibition of mCRPC by new-generation anti-androgens such as abiraterone, which blocks androgen biosynthesis, or the AR antagonist enzalutamide, which prevents the receptor's nuclear translocation and DNA binding, provides a clinically effective treatment strategy for a patient subgroup, although therapeutic responses are non-durable [5–8]. Synthetic lethality from the inhibition of poly (ADP-ribose) polymerase (PARP) provides further clinical benefits to mCRPC patients who carry defective DNA repair genes [9].

The PI3-kinase/AKT/mTORC1 oncogenic axis is activated in ~40% primary prostate cancer and ~70% mCRPC, ascertained on the basis of whole-genome exome profiling of tumor samples from several hundred patients [10]. The serine-threonine kinase activity of the mTORC1 complex relays the functional impact of an activated PI3-kinase/AKT axis by promoting cell growth through multiple downstream effectors which, among others, enhance protein synthesis from increased ribosome biogenesis and messenger RNA translation under nutrient-rich conditions; promote lipid biogenesis; and inhibit autophagy [11–13]. The multi-protein complex mTORC1 contains two molecules each of mTOR, a serine-threonine kinase, and raptor (regulatory-associated protein of mTOR), the mTOR-interacting protein that stabilizes the mTOR dimer and promotes mTOR's substrate specificity [13]. Other components of the complex are: the DEP-domain containing daptor, the scaffold protein mLST10/GβL and the mTOR inhibitor PRAS40, which is a 40kDa proline-rich AKT substrate. The TSC2/TSC1 tuberous sclerosis complex, acting downstream of AKT, negatively regulates mTORC1 by inhibiting the GTPase activity of Rheb (Ras Homolog Enriched in Brain), which is a positive regulator of mTORC1. The mTORC1 complex is a hub for signal convergence from numerous positive and negative regulatory cascades. TSC2/TSC1 inhibition due to AKT-mediated TSC2 phosphorylation at serine-939 and threonine-1462 leads to mTORC1 activation, whereas mTORC1 inhibition and consequent cell growth arrest in response to nutrient deprivation or oxidative stress is caused by activation of AMP-activated kinase (AMPK) and a phosphorylation-dependent cascade that ensues. TSC2 phosphorylation at serine-1387 by AMPK stimulates TSC2/TSC1 activity, thus inhibiting mTORC1. Raptor phosphorylation at serine-722 and Ser-792 by AMPK also inactivates mTORC1 [14].

Activation of the PI3-kinase (PI3K) axis from PTEN deficiency suppressed AR transcriptional output in murine models of prostate cancer [15, 16]. However, in clinical trials PI3K pathway blockers did not impede mCRPC growth, which is at least partly due to AR's enhanced stability and activity as a result of its phosphorylation by reactivated HER family kinases [16]. Reciprocally, AR inhibition in PTEN-null prostate cancer caused AKT activation due to destabilization of the AKT phosphatase PHLPP, which resulted from reduced AR-targeted expression of FKBP5, a chaperone protein for PHLPP. Although an AR antagonist in combination with a PI3K/AKT pathway blocker curbed castration-resistant prostate cancer in Pten-null mice and human prostate tumor xenografts [16, 17], a combined regimen of these inhibitors showed low activity in a clinical trial [18]. A dual-acting AR/mTORC1 inhibitor may show the desired clinical efficacy that is absent in combination therapy.

Herein we provide the first evidence that salinomycin, a potassium ionophore and a human cancer stem cell inhibitor, can lower AR expression and activity, reduce protein levels of CYP17A1 and HSD3β1, the critical regulators of the androgen biosynthesis pathway, and block mTORC1 activity in PTEN-deficient prostate cancer cells. These molecular changes are associated with reduced proliferation of castration-sensitive and castration-resistant prostate cancer cells *in vitro* and inhibition of prostate tumor growth *in vivo* in xenograft tumor models. Loss of serine-81 AR phosphorylation preceded total AR reduction in salinomycin-treated cells. Inhibition of mTORC1 was associated with enhancement of AMPK-mediated phosphorylation of TSC2 and raptor, as well as reduction of TSC2 phosphorylation by AKT. Our results suggest that salinomycin may be clinically active as monotherapy against advanced prostate cancer.

## RESULTS

### Salinomycin-induced cytostasis, apoptosis and autophagy

**Figure 1:**
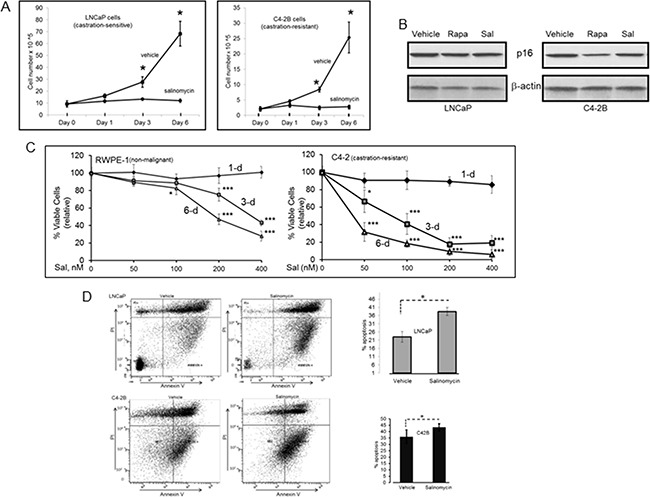
Salinomyin inhibited proliferation and increased apoptosis of prostate cancer cells, but did not induce cellular senescence **A.** Cell numbers at day-1, -3 and -6 post-treatment. Each point is average of three biological replicates; Cell number for an individual experiment is average from duplicate wells. At day-0, cells were seeded at equal numbers in all wells. Plots show viable cells relative to the starting number of seeded cells. * p<0.05. **B.** p16 western blotting to assess cellular senescence. **C.** RWPE-1 and C4-2 viable cells over 1-, 3- and 6-day periods at 50 nM, 100 nM, 200 nM and 400 nM Sal. Each data point is average of three biological replicates. * p<0.05; *** p<0.001. **D.** Early apoptotic cells. Dual parameter dot plots combined AnnexinV-Fluorescein isothiocyanate (FITC) and propidium iodide (PI) fluorescene. Viable cells (AnnexinV^−^PI^−^), lower left quadrant; early apoptotic cells (AnnexinV^+^PI^−^), lower right quadrant; upper right and left quadrants, late apoptotic/necrotic cells. Bar graphs show early-apoptosis cell numbers at 3-day post-treatment; *p<0.05. Sal: salinomycin; Rapa: rapamycin.

Salinomycin inhibited cell proliferation for AR-expressing LNCaP (castration-sensitive) and C4-2B (castration-resistant) human prostate cancer cells (Figure 1A). Inhibition was not due to cellular senescence, since p16, a cell cycle inhibitor and marker for senescent cells, was not induced (Figure 1B). The mTORC1 inhibitor rapamycin, which is known to reduce prostate cancer cell proliferation, also did not cause p16 induction. The cancer cells were significantly more sensitive to salinomycin than RWPE-1 non-malignant prostate epithelial cells (Figure 1C). Relative to the initial number of seeded cells, the drug at 200 nM reduced RWPE-1 cells ~20% and ~50% after 3-day and 6-day incubation, respectively. In contrast, the same concentration of salinomycin reduced castration-resistant C4-2 cells >80% on day-3 and >90% on day-6. Significantly less inhibition of RWPE-1 cells than C4-2 cells by 400 nM salinomycin was also observed over 3- and 6-day treatment periods. The inhibition is at least partly due to cytostasis, since gene expression profiling of PC3 prostate cancer cells indicated that salinomycin may induce cell cycle arrest [20]. Cytostasis is further indicated by our result that salinomycin reduced the growth of xenograft tumors without ablating the pretreatment tumor mass (described later in Figure 7).

AnnexinV+PI− cells, indicating early apoptosis, increased significantly after cells were treated with the drug for 3 days (Figure 1D). The modest increase in apoptosis was attributed to the low salinomycin concentration (400 nM) for the study in Figure 1D. Robust cleavage of PARP-1 and procaspase-3 in C4-2 cells (indicating apoptosis) was observed at 1 uM salinomycin but not at 400 nM (data not shown). In an earlier study, we documented PC-3 cell apoptosis by 1 uM salinomycin [19].

**Figure 2:**
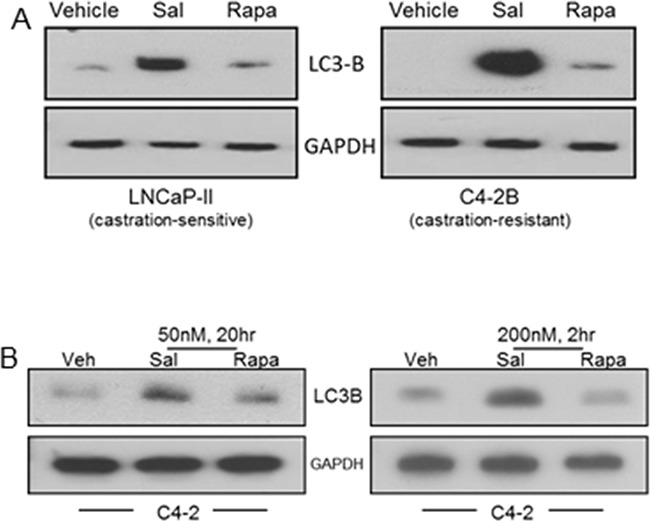
Differential autophagy induction in prostate cancer cells by salinomycin vs. rapamycin, revealed from LC3B induction **A.** LNCaP-II and C4-2B cells, treated 20 hours with 400 nM Sal or 50 nM Rapa. **B.** C4-2 cells, 20hr or 2hr treatment with either drug at 50 nM or 200 nM, respectively.

Autophagy induction by salinomycin was revealed from elevated LC3B levels in LNCaP-II (an LNCaP variant) and C4-2B cells (Figure 2A). Autophagosome-associated LC3B is the phosphatidyl ethanolamine-conjugated form of the cytosolic LC3 (microtubule-associated light chain3) and a marker for cellular autophagy. At equivalent doses (50 nM or 200 nM), autophagy in C4-2 cells was induced more robustly by salinomycin than rapamycin (Figure 2B).

### Inactivation of mTORC1

**Figure 3:**
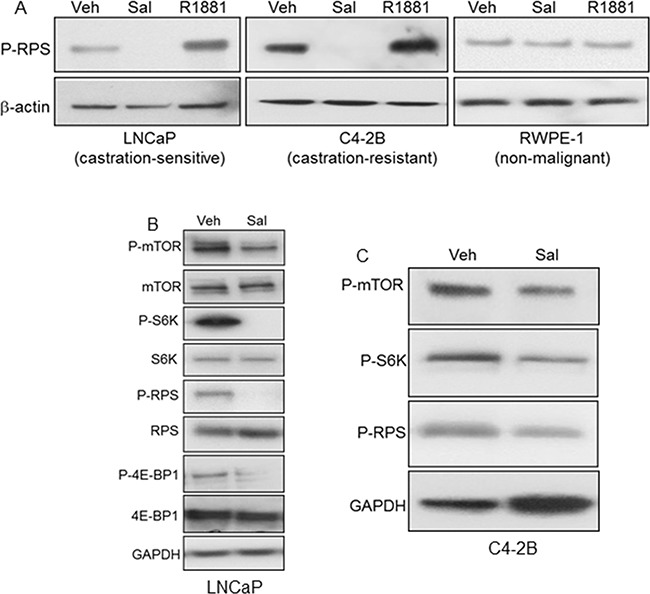
Inhibition of mTORC1 by salinomycin in LNCaP and C4-2B cells **A.** Phospho-S6 ribosomal protein (P-RPS) in cells treated for 20 hours with Sal, 400 nM or R1881, 1nM. **B, C.** Reduced phosphorylation of mTOR, S6 kinase-1 (S6K), RPS and 4E-BP1.

Since autophagy is negatively regulated by mTORC1, we examined the effect of salinomycin on mTORC1 activity by analyzing mTOR phosphorylation as well as phosphorylation of the mTORC1 substrate S6 kinase-1 (S6K) and the downstream effector ribosomal protein S6 (RPS), which is an S6K substrate (Figures 3A-C). Marked decrease of phospho-RPS in salinomycin-treated LNCaP and C4-2B cells demonstrated mTORC1 inhibition (Figure 3A). P-RPS levels did not differ significantly for control *versus* salinomycin-treated RWPE-1 cells (Figure 3A). The enhanced P-RPS signal by the synthetic androgen R1881 (Figure 3A) is consistent with earlier reports showing stimulation of mTORC1 in LNCaP cells by the androgen-activated AR [21, 22]. Inhibition of mTORC1 by salinomycin in AR-negative PC3 and DU145 prostate cancer cells was reported [23]. Dose- and time-dependent P-RPS reduction by salinomycin is shown as supplement data (Supplementary Figure S1). P-RPS decreased markedly in LNCaP cells by treatment with 300 nM salimomycin for 2 hours and at 2-fold lower drug concentration (150 nM), P-RPS levels decreased markedly by 6 hrs. (Supplementary Figure S1A, S1B). P-RPS was undetectable in C4-2 cells at 6 hours upon incubation with 400 nM salinomycin (Supplementary Figure S1C). P-RPS reduction by 50 nM rapamycin served as a control (Supplementary Figure S1C). Other inhibitors of prostate cancer cells, such as the plant lignin deoxy-podophyllotoxin (DPT, ref [24]), vitamin D receptor agonist EB1089 and liver X receptor agonist T090198, did not significantly reduce P-RPS in PC-3 and C4-2B cells (Supplementary Figures S2-A, -B). Reduction of phospho-mTOR and phospho-S6K provided direct evidence for mTORC1 inactivation (Figures 3-B, C). Decreased phosphorylation of 4E-BP1, the eukaryotic initiation factor 4E binding protein and an S6K substrate, further demonstrated mTORC1 inactivation (Figure 3B).

### Inhibition of AR mRNA and protein expression, AR Ser-81 phosphorylation and AR-mediated transactivation -- a contrast to rapamycin

**Figure 4:**
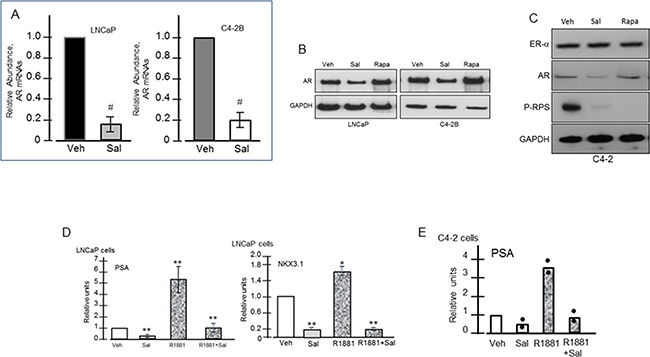
Reduced expression of AR and AR-targeted genes in response to salinomycin Cells were treated with Sal (400 nM) for 20 hrs. **A.** QRT-PCR of AR mRNAs; # p<0.05. **B.** Western blot assay of AR. **C.** ERα, AR, P-RPS levels in C4-2 cells. **D, E.** PSA and Nkx3.1 mRNA levels in LNCaP (D, E) and C4-2 (D) cells; * p<0.05; ** p<0.01. Each bar graph is the average of two independent assays. For E, points represent individual values.

Salinomycin reduced AR mRNA and protein levels in LNCaP and C4-2B cells (Figures 4A & 4B). Reduction of AR mRNAs was detected within 6 hours of drug treatment (Supplementary Figure S4C). Estrogen receptor-α (ER-α) expression was unchanged, indicating a selective effect of the drug on AR (Figure 4C). Rapamycin, on the other hand, elevated AR levels (Figure 4B), which is consistent with reports that rapamycin enhanced AR stability and transactivation activity [15, 16, 25, 26]. Androgen-activated AR target gene expression was inhibited by salinomycin, since PSA and NKX3.1 mRNAs declined 5- to 7-fold in LNCaP cells and PSA mRNAs declined ~3.5-fold in C4-2B cells (Figures 4D, 4E). Basal expression of PSA and NKX3.1 mRNAs was also reduced, possibly due to interference with the ligand-independent transcription activity of AR.

**Figure 5:**
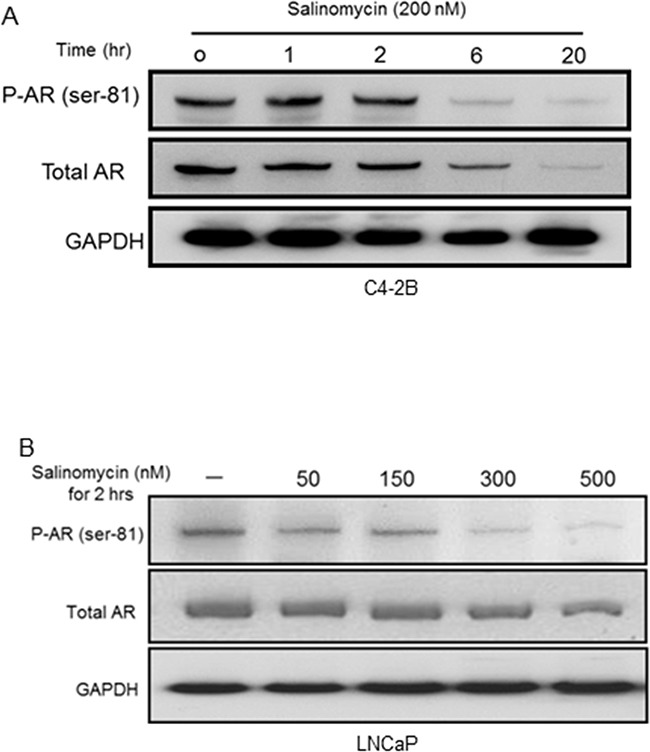
Inhibition of AR phosphorylation at serine-81 **A.** PhosphoAR-Ser81 in C4-2B cells, which were incubated with Sal for increasing time periods. The membrane was probed for phosphoAR-Ser81, and re-probed (after stripping) for total AR. **B.** PhosphoAR-Ser81 levels in LNCaP cells.

AR phosphorylation at serine-81, a metric of its nuclear activity, was inhibited by salinomycin -- the inhibition preceding total AR reduction (Figure 5). In C4-2B cells, 6hr incubation with 200 nM salinomycin markedly diminished the phospho-AR (Ser-81) signal without a proportional reduction of total AR (Figure 5A). The phospho-AR(Ser-81) level declined in LNCaP cells within 2 hours by salinomycin as low as 50 nM, and the phospho-AR signal dropped precipitously at 2hr incubations with 300 nM and 500 nM salinomycin – the conditions which did not significantly reduce total AR (Figure 5B). In C4-2 cells also, AR phosphorylation at Ser-81 was reduced prior to AR reduction, while Ser-81 phosphorylation was not affected by rapamycin (Supplementary Figure S3). From these results we conclude that inhibition of AR's Ser-81 phosphorylation preceded total AR reduction.

### Reduction of CYP17A1 and HSD3β1 levels

**Figure 6:**
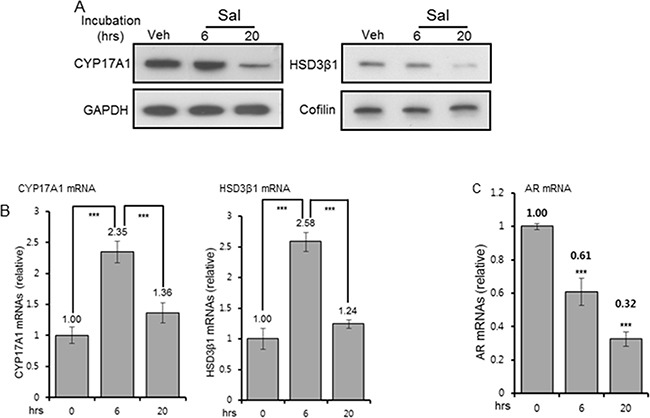
Reduction of CYP17A1, HSD3β1 and AR levels **A.** CYP17A1 and HSD3β1 protein levels in C4-2 cells treated for 6 and 20 hours with 400 nM Sal. GAPDH (37kDa) and cofilin (20kDa) were internal controls for CYP17A1 (57kDa) and HSD3β1 (42kDa), respectively. For each, the same blot, cut in two segments, was used to probe the antigen of interest and internal control antigen. Control C4-2 cells were vehicle-treated for 20 hours. **B, C.** QRT-PCR of CYP17A1, HSD3β1(B), and AR (C) mRNAs, normalized to constant GAPDH mRNAs. Each bar graph is average of three biological replicates. For technical replicates, values from duplicate wells were averaged. *** p<0.001.

CYP17A1 (cytochrome P450 lyase/hydroxylase) and HSD3β1 (hydroxysteroid dehydrogenase-3β1) are key regulators of the androgen biosynthesis pathway and thus, the intratumoral androgen level. Reduced expression of both enzymes in C4-2 cells was observed upon 20hr incubation with salinomycin. No change in CYP17A1 and HSD3β1 was detected at 6 hour (Figure 6A). GAPDH (37kDa) and cofilin (19-20kDa) were internal controls for CYP17A1 (57kDa) and HSD3β1(42kDa), respectively. The salinomyin effect was selective since levels of SULT2B sulfotransferase, the androgen-metabolizing enzyme which is frequently reduced in human prostate cancer [27], did not change (Supplementary Figure S4C). The mRNAs for CYP17A1 and HSD3β1 increased more than 2-fold after 6hr drug incubation, although their protein levels at 6 hour were unchanged, and mRNAs for both enzymes reverted to basal levels by 20 hours (Figure 6B). Induction of CYP17A1 and HSD3β1 mRNAs at the earlier time point possibly reflects compensatory responses to altered AR mRNAs, which were reduced ~40% at 6hr drug incubation and ~70% by 20 hours (Figure 6C). Cq values, determined from quantitative RT-PCR assay, revealed that CYP17A1 and HSD3β1 mRNAs were expressed at much lower levels in these cells compared to AR mRNAs. CYP17A1 protein reduction by salinomycin was detected in multiple cell lines under conditions when rapamycin had no effect (Supplementary Figure S4A). Reduction of HSD3β1 in C4-2 cells in a dose and time-dependent manner was further evidence that salinomycin negatively regulates this enzyme (Supplementary Figure S4B). It remains to be determined whether loss of stability as a consequence of salinomycin-mediated endoplasmic reticulum stress and/or mitochondrial membrane depolarization had led to the reduction of these microsomal enzymes. In any event, we conclude that salinomycin can potentially lower tumor cell androgen levels by inhibiting CYP17A1 and HSD3β1, thereby further restricting AR signaling.

### Inhibition of prostate tumor xenografts

**Figure 7:**
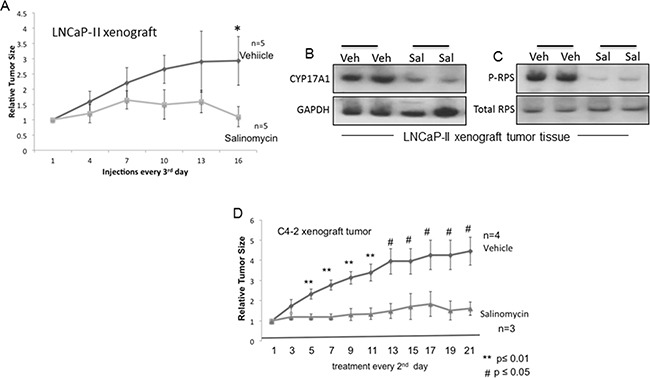
Inhibition of prostate tumor xenografts by salinomycin **A.** Growth curves for LNCaP-II xenografts in nude male mice treated with vehicle or salinomycin. Mice received i.p. injections of salinomycin or vehicle every 3^rd^ day; n=5. * p ≤ 0.04. **B, C.** CYP17A1 (B) and phospho-RPS (C) levels in LNCaP-II xenografts, showing data from two individual mice for control and experimental groups. **D.** Growth rates of C4-2 tumor xenografts. Salinomycin (or vehicle) was delivered via oral gavage every 2^nd^ day. ** p ≤ 0.01; #p ≤ 0.05.

Mice carrying xenograft tumors of LNCaP-II (Figure 7A) and C4-2 (Figure 7D) cells were randomized to vehicle and salinomycin treatment groups after tumors in individual mice had reached ~ 200-250 mm^3^. For LNCaP-II xenografts, intraperitoneal (i.p.) injections of salinomycin (5 mg/Kg body weight) or vehicle (DMSO) were administered every 3^rd^ day till day-16, after which tumors were harvested. Tumor growth rate declined significantly for the salinomycin group after 6 injections compared to the vehicle control p<0.04, n=5 (Figure 7A). Since tumor growth rates varied mouse-to-mouse, the tumor size at any indicated day was normalized to the tumor size for the same mouse on the 1^st^ day of treatment. Overt toxicity from the drug was likely absent, since the two groups of mice did not differ significantly in body weights (data not shown) and prostate histology (Supplementary Figure S5). Salinomycin reduced CYP17A1 and P-RPS levels in LNCaP-II xenografts (Figures 7B, 7C).

For C4-2 tumor xenografts, salinomycin was delivered every 2^nd^ day till day-21 using oral gavage (Figure 7D). C4-2 xenografts were castration-resistant, since C4-2 cells proliferated in culture in the absence of androgen, and C4-2 xenograft tumors developed in castrated mice. Reduction of tumor volumes for the salinomycin group after 2 injections (day-5) was significant compared to the vehicle group, and lower tumor volumes of the salinomycin group persisted throughout the treatment period. Thus, irrespective of the route of drug delivery, salinomycin reduced tumor burden in castration-sensitive and castration-resistant xenografts.

### Mechanism underlying mTORC1 inhibition

**Figure 8:**
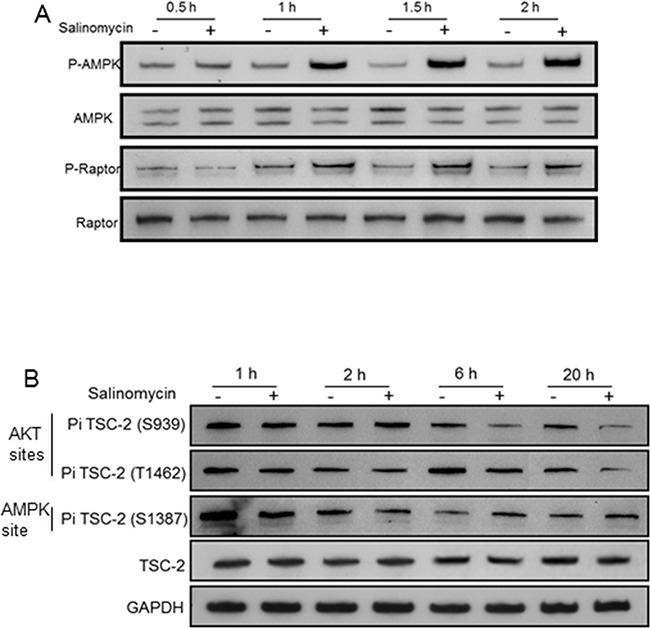
Salinomycin-induced changes in the phosphorylation of AMPKα, raptor and Tuberin/TSC2 C4-2 cells were incubated with 100 nM Sal and analyzed for **A.** 62 kDa AMPKα/phospho-AMPKα(Thr-172) and 150 kDa raptor/phospho-raptor(Ser-792). **B.** 200 kDa TSC2/phospho-TSC2 at Ser-939/Thr-1482 and at Ser-1387.

Reactive oxygen species (ROS) levels were elevated by salinomycin in prostate cancer cells [19]. Since oxidative stress activates AMPK [28, 29], Thr-172 phosphorylation of AMPK-α (a measure of AMPK activation) was examined in C4-2 cells. The phospho-AMPK-α(Thr-172) level increased by salinomycin within 1 hour, and it remained elevated at 1.5 hours and 2 hours (Figure 8A). AMPK activation led to increased raptor phosphorylation at Ser-792 (an AMPK site) at 1 hour, and phospho-raptor increased further at 1.5 and 2 hours (Figure 8A), Un-phosphorylated raptor levels did not change (Figure 8A). TSC2 phosphorylation at Ser-1387, which is an AMPK-targeted site, increased within 6 hours (Figure 8B). Phosphorylation of TSC2 and raptor by AMPK is known to elevate TSC2/TSC1 activity and reduce mTOR's kinase function, respectively [14, 30]. Thus, the activated AMPK-TSC2 and AMPK-raptor axis led to mTORC1 inactivation.

Reduced TSC2 phosphorylation at Ser-939 and Thr-1462 (two AKT-targeted sites) within 6hr drug treatment (Figure 8B) also contributed to mTORC1 inactivation, since AKT-mediated TSC2 phosphorylation at Ser-939/Thr-1462 lowered TSC2/TSC1 activity by destabilizing TSC2-TSC1 interaction [31]. Although mTORC1 inactivation prevented feedback inhibition of PI3K by p70S6K, and thus increased PI3K activity, inhibitory phosphorylation of TSC2 by PI3K-activated AKT was blocked by salinomycin thereby preventing reduction of TSC2/TSC1 activity. No change occurred in PRAS40, its phospho-modified form (P-PRAS40), Rheb and FKBP12 levels (Supplementary Figure S6). FKBP12, in complex with rapamycin, causes allosteric inhibition of mTOR kinase [32].

## DISCUSSION

The present study identified salinomycin as the first example of a dual-acting inhibitor of AR and mTORC1. Salinomycin, a polyether antibiotic, inhibited PTEN-null castration-sensitive and castration-resistant prostate cancer cells for expansion *in vitro* and in tumor xenografts, and prevented a compensatory negative crosstalk between AR and mTORC1, which otherwise would have led to reciprocal activation of the two oncogenic pathways, causing activation of one when the other is blocked. This compensatory interplay is believed to have contributed to the low activity of a combined regimen of everolimus (mTORC1 inhibitor) and bicalutamide (AR antagonist) against metastatic prostate cancer in a phase II trial [18]. This clinical finding, however, differed from preclinical results that documented inhibition of Pten-null prostate cancer when pharmacologic inhibitors of AR and PI3K/mTORC1 or AKT activity were used concurrently [16, 17, 33].

**Figure 9:**
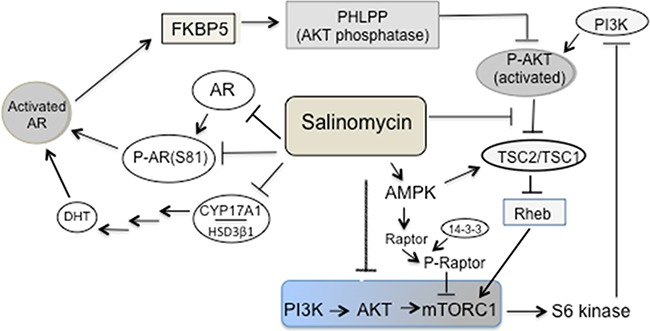
Multi-pronged targeting of the AR and PI3K/mTORC1 axis by salinomycin – a schematic illustration Reduction of AR expression, AR Ser-81 phosphorylation as well as CYP17A1 and HSD3β1 expression led to the inhibition of AR activity. Salinomycin inhibited the PI3K→AKT→mTORC1 axis due to 1) activation of AMPK, which in turn induced raptor phosphorylation and binding of 14-3-3 to phospo-raptor would dampen mTOR kinase function (14). Activated AMPK also augmented TSC2/TSC1 activity by mediating stimulatory phosphorylation of TSC2 at Ser-1387; 2) inhibition of the AKT→TSC2 axis, since inhibitory phosphorylation of TSC2 at Ser-939/Thr-1482, catalyzed by AKT, was reduced. Elevated TSC2/TSC1 activity led to mTORC1 inactivation. Salinomycin-mediated targeting of the AKT→TSC2 axis mitigated the impact of AKT activation caused by enhanced PI3K activity, which resulted from the loss of feedback repression.

Figure 9 summarizes our results demonstrating multi-faceted targeting of the AR and mTORC1 axis by sub-micro molar salinomycin. Inhibition of AR Ser-81 phosphorylation -- a modification that promotes AR's stability; nuclear localization; chromatin binding and transcription activity, preceded AR mRNA and protein reduction. Reduced CYP17A1 and HSD3β1 expression is expected to further impair androgen-induced AR activity by inhibiting de novo androgen production. Information as to whether DHEA and androgen levels would also decline in the presence of salinomycin awaits future analysis. Enhanced raptor and TSC2 phosphorylation by AMPK led to decreased phospho-mTOR and thus, mTORC1 inactivation, as well as reduced phosphorylation of p70S6K and the downstream targets RPS and 4E-BP1. Salinomycin also blocked inhibitory phosphorylation of TSC2 by AKT, even though AKT was activated due to enhanced PI3K activity, which was caused by abrogation of S6K-mediated feedback repression. Of note is the reported finding that the serine protease kallikrein 4 (KLK4), which promotes prostate cancer growth, activated both the AR and mTORC1 axis due to negative interplay of KLK4 with the transcription factor PLZF (promyelocytic leukemia zinc finger) [34]. The effect of salinomycin on KLK4-PLZF interaction may provide additional insights into the multiple pathways that are targeted by this drug to cause prostate cancer inhibition.

Increased RPS phosphorylation by the synthetic androgen R1881 is consistent with previous reports that androgen stimulated mTORC1 in LNCaP cells due to induction of nutrient transporters, which would increase nutrient availability [21, 22]. Robust autophagy induction by salinomycin was at least partly due to inhibition of mTORC1, which negatively regulates autophagy, and also due to activated AMPK, which promotes autophagosome formation [14, 35–39]. Under nutrient-rich conditions, mTORC1 limits autophagy to counter lysosomal engulfment and degradation of cellular organelles and other cytosolic contents by mediating inhibitory phosphorylation of the ULK1-Atg13-FIP-200 complex, an obligatory component of autophagosomes [35]. On the other hand, AMPK stimulates phosphorylation of the ULK-1 complex, which is essential for autophagosome formation [39]. Autophagy by rapamycin was less prominent than by salinomycin (Figure 2A, B). This is likely due to a lack of AMPK activation by rapamycin. Endoplasmic reticulum (ER) stress may additionally contribute to salinomycin-induced autophagy in prostate cancer cells, similar to what was reported for lung cancer cells [40]. Although autophagy is generally considered to be a pro-survival response, in certain contexts autophagy can induce cancer cell death or cytostasis [41, 42]. It remains to be determined whether salinomycin-induced autophagy is linked to non-apoptotic, autophagy-induced cell death (autosis) or cytostatic autophagy, and how the salinomycin effect on prostate cancer may be modulated by agents that inhibit autophagy.

Impaired AR signaling by salinomycin due to interference with AR's Ser-81 phosphorylation, AR mRNA and protein reduction and inhibition of AR transcriptional readout – all of which are novel findings in the present study- contrasts rapamycin-mediated increase in the AR level and activity, which likely contributed to the clinical failure of everolimus against castration-resistant prostate cancer [18, 43]. CYP17A1 and HSD3β1 reduction is expected to limit de novo androgen production, which would also lower AR activity. Ser-81 is the most abundantly phosphorylated amino acid among various androgen-induced serine phosphorylation sites of AR [44], and phospho-AR(Ser-81) can promote prostate cancer cell growth and enhance AR's stability, chromatin binding and transcriptional activity – all indicating an important role for this phospho modification in human prostate cancer [45–48]. Loss of AR's Ser-81 phosphorylation preceded loss of total AR, (Figures 5A, B). The kinases CDK9, CDK1 and CDK5 regulate Ser-81 phosphorylation [44–46], and inhibition of these CDKs or enhanced expression/activity of a phosphatase may have impaired Ser-81 phosphorylation. A Ser-81-targeting phosphatase is yet to be identified; to this end, a protein phosphatase-1 mediating de-phosphorylation at AR-Ser650 was reported to enhance AR's stability and nuclear import [49]. Since Ser-81 de-phosphorylation was an early event, preceding reduction of AR, CYP17A1 and HSD3β1, insights into phosphorylation/de-phosphorylation dynamics at Ser-81 may uncover new target(s) for AR inhibition.

ERK1/2 Map kinase was activated by salinomycin and rapamycin (Supplementary Figure S7A), which is an expected outcome of mTORC1 inhibition since PI3K, relieved of S6K-imposed feedback repression, would stimulate the RAF→MEK→ ERK1/2 cascade. Similarly, ERK1/2 was activated by a rapamycin analog in breast cancer cells [50]. Enhanced phosphorylation of AKT and GSK (AKT substrate) in salinomycin-treated LNCaP cells (Supplementary Figure S7B) also proved elevated PI3K activity. Combining everolimus with a MEK1/2 inhibitor was more effective than either inhibitor alone in curbing MCF7 breast cancer xenograft growth [50]. How a salinomycin-MAP kinase inhibitor combination would compare with salinomycin alone in targeting prostate tumor growth will be addressed in the future.

AMPK activation by salinomycin (Figure 8A) is in keeping with our previous finding that salinomycin elevated reactive oxygen species (ROS) in prostate cancer cells [19], and reports by others that ROS activates AMPK [28, 29]. In salinomycin-treated cells, AMPK catalyzed raptor phosphorylation (at Ser-792, Figure 8A), and TSC2 phosphorylation (at Ser-1387, Figure 8B). Phosphorylation of raptor and TSC2, as downstream effectors of the ROS-AMPK axis, reduced mTORC1 activity since i) raptor phosphorylation at Ser-722/Ser-792 can promote its binding to 14-3-3, which would attenuate the Ser-Thr kinase function of mTOR [14]; and ii) stimulatory phosphorylation of TSC2 at Ser-1387 can enhance the TSC2/TSC1 tuberous sclerosis complex activity, which is a negative regulator of mTORC1 [14]. Enhancement of TSC2/TSC1 activity was also due to interference with the AKT-mediated inhibitory phosphorylation of TSC2 at Ser-939/Thr-1462. This mitigates the impact of AKT activation due to increased PI3K activity, which is a consequence of abrogated feedback repression. AMPK activation and raptor phosphorylation were detected within 1 hour of drug treatment, whereas increased TSC2 phosphorylation at Ser-1387 and reduced TSC2 phosphorylation at Ser-939/Thr-1462 were detected after 2hrs -- changes being more prominent at 20 hours (Figure 8B). Delayed kinetics for TSC2 phosphorylation changes is possibly due to low (100 nM) salinomycin concentration (Figure 8), since at 100 nM, the drug markedly reduced P-RPS after 6hr incubation, but not at 2 hour (Supplementary Figure S1C). Also, TSC2 phosphorylation kinetics may be slower than that for raptor. We conclude that a stimulated ROS-AMPK axis and attenuated AKT-TSC2 axis contributed to mTORC1 inactivation.

In summary, salinomycin is the first example of a dual-acting AR/mTORC1 inhibitor, which can inhibit castration-sensitive and castration-resistant human prostate cancer cells *in vitro* and in xenograft models. The drug did not cause overt toxicity, since tumor growth inhibition was not associated with changes in prostate histology (Supplementary Figure S5) and mouse body weights. The inhibition was associated with decreased AR levels and activity, reduction of CYP17A1, HSD3β1 and mTORC1 inactivation. Elevated TSC2/TSC1 activity due to the stimulated AMPK→TSC2 and attenuated AKT→TSC2 axis, as well as activation of the AMPK→raptor axis, culminated in mTORC1 inactivation.

Cancer stem cells and therapy-refractive bulk cancer cell populations in xenografts of various human cancers were eliminated and metastasis was prevented by salinomycin [51–53]. A few case reports documented partial regression of various solid tumors by salinomycin delivered in conjunction with conventional chemotherapy, and side effects from salinomycin were temporary and minor [54]. These findings, and our present work, provide a framework for future study to explore the efficacy of salinomycin (or its analog) as a stand-alone agent against clinical progression of prostate cancer.

## MATERIALS AND METHODS

### Reagents, cell lines

Salinomycin, Sigma-Aldrich (St Louis, MO); rapamycin, LC Laboratories (Woburn, MA); DPT, T0901317 (Cayman Chemical, Ann Arbor, MI); EB1089 (Medchemexpress USA, Monmouth Junction, NJ). LNCaP, RWPE-1 and PC-3 are from American Type Culture Collection (Manassas, VA); LNCaP-II, C4-2, C4-2B, ViroMed Laboratories Inc. (Hopkins, MN). RWPE-1 cells were cultured in keratinocyte medium plus 5% fetal bovine serum (FBS), supplemented with penicillin and streptomycin. Other cell lines were in RPMI 1640 with 5% FBS plus penicillin/ streptomycin

### Cell proliferation, apoptosis

Viable cells (Trypan blue excluded) were counted in an automated cell counter (Invitrogen, Carlsbad, CA). Cell number for each experiment was average from duplicate wells. Cells seeded at 10^6^cells/well (6-well dishes) were treated with vehicle or salinomycin at 48-hour post-seeding. Each point is average of three biological replicates. Annexin V-and propidium iodine(PI)-stained cells were analyzed by flow cytometry. Cells were stained using Annexin V Apoptosis Detection Kit APC (Cat # 88-8007-72, Affymetrix-eBioscience, San Diego, CA). Samples were acquired on LSRII (BD Bioscience, San Jose, CA) within 30 minutes of conclusion of Annexin V staining. The Diva (BD Bioscience, San Jose, CA) or FlowJo (Tree Star, Ashland, OR) software was used for analysis.

### Antibodies

Cell Signaling Technology (Danvers, MA) was the source for antibodies to mTOR/ phospho-mTOR, Ser2448(D9C2); AKT1/phospho-AKT1, Thr308(#9275); Tuberin (TSC2)/phospho-Tuberin (TSC2), Thr1462(5B12); p70S6Kinase/phospho-p70S6 Kinase, Thr389 (#9205); GSK3α/β- phospho-GSK3β, Ser9 (#9322); S6 Ribosomal Protein/phospho-S6 Ribosomal Protein, Ser235/ 236(#2211); 4E-BP/phospho-4E-BP, Thr37/46(#9459); AMPKα/phospho-AMPKα, Thr172(40H9); Raptor/phospho-Raptor, Ser792 (#2083); PRAS40/phospho-PRAS40, Thr246(D4D2); ERK1/2/phospho-ERK1/2, Thr202/Tyr204 (D13.14.4E); Rheb (E1G1R); LC3B (D11). Antibodies to AR(N-20), FKBP12(N-19), GAPDH, β-actin and cofilin (FL-166: sc-33779) from Santa Cruz Biotech (Dallas, TX); Antibody to phospho-AR, Ser-81(#07-1375) from EMD-Millipore (Billerica, MA). Antibodies to: CYP17A1 (EPR 6293) from GeneTex (Irvine, CA); HSD3β1 (ARP 41821-P050) from Aviva Systems Biology (San Diego, CA) and p16 (2D9A12, ab54210) from Abcam (Cambridge, MA).

### QRT-PCR, western blotting

Total cell RNAs were isolated using TRIZOL® and converted to cDNAs for QRT-PCR analysis using previous conditions [27]. PCR Primers: AR: ^5′^AAGA-CGCTTCTACCAGCTCACCAA-sens.e, ^5′^TCCC AGAAAGGATCTTGGGCACT-antisense; PSA: ^5′^TG ACCAAGTTCATGCTGTGT-sense, ^5′^GTCATTTCCAAGG TTCCAAG-antisense; NKX3.1: ^5′^AC-TTGGGGTCTTATC-sense, ^5′^CTTCTGCGGCTGCTTAGGG-antisense; CYP17A 1: ^5′^ACCTGGAGG TGCCAGATGAT-sense, ^5′^GGCG CACCTTGATCTTCACT-antisense; GAPDH internal control: ^5′^GA-CAGGATGCAGAAGGAGAT-sense, ^5′^TT GCTGATCCACATCTGCTG-antisense. A validated primer set, Bio-Rad (Cat#10025636) was used for qPCR of HSD3β1 cDNAs. QPCR was performed using CFX384 Real Time PCR machine (Bio-Rad, Hercules, CA).

For protein analysis, harvested cells were taken up in RIPA buffer (50 mM Tris-HCl pH 8.0, 150 mM NaCl, 0.1% Triton X-100, 0.5% sodium deoxycholate, 0.1% SDS) containing protease inhibitors (EDTA free cocktail tablet, Roche, cat#873-580-001 plus 1mM PMSF). For phospho-protein analysis, protein phosphatase inhibitor (Halt™ cat# 78440, Thermo Fisher Scientific) was added to RIPA buffer containing protease inhibitors. Cells were sonicated (30 sec, 4°C) using Sonic Dismembrator, setting 5 (Fisher Scientific, Model 60). Supernatants of lysed cells were analyzed by Western blotting after 10% SDS-PAGE [27]. Two segments, cut from the same western blotted membrane were antibody probed for CYP17A1 (57kDa) and GAPDH (37kDa). A second membrane was similarly segmented and then probed for HSD3β1(42kDa) and cofilin (19-20kDa). With this strategy, we avoided the use of stripped membranes for sequential probing of internal controls (GAPDH and cofilin).

### Tumor xenografts and treatment

Tumor xenografts were produced subcutaneously at the right flank of athymic nude mice (6-wk male, Charles River Laboratory, Wilmington, MA) with cells at 3 × 10^6^ cells, mixed 1:1 (v/v) with matrigel (20 mg/ml, Corning). Treatment started at ~200-250 mm^3^ of palpable tumor. Tumor volume=½(length × width^2^); length=maximum longitudinal diameter and width=maximum transverse diameter, measured with a caliper. Tumors with an adjacently placed ruler were photographed for record keeping. Mice received salinomycin (5mg/kg in DMSO, stock solution diluted in sterile water) and vehicle (DMSO, diluted with sterile water) by i.p. injections (LNCaP-II xenografts) or oral gavage (C4-2 xenografts). Tumor lysates were prepared in RIPA buffer. Protocol approved by the Institutional Animal Care and Use Committee was used.

### Statistical analysis

Data were analyzed using SPSS 15 software. Student's t-test was used; data presented as mean ± SEM; p < 0.05 is significant.

## SUPPLEMENTARY FIGURES


