# Supplementary material for: Dual targeting of androgen receptor and mTORC1 by salinomycin in prostate cancer
Source: Oncotarget. 2016 Aug 19;7(38):62240–54. doi: 10.18632/oncotarget.11404 (PMC5308723; doi:10.18632/oncotarget.11404)
Supplement: Supplementary file 1 [file oncotarget-07-62240-s001.pdf]

Dual targeting of androgen receptor and mTORC1 by salinomycin in prostate cancer

SUPPLEMENTARY FIGURES

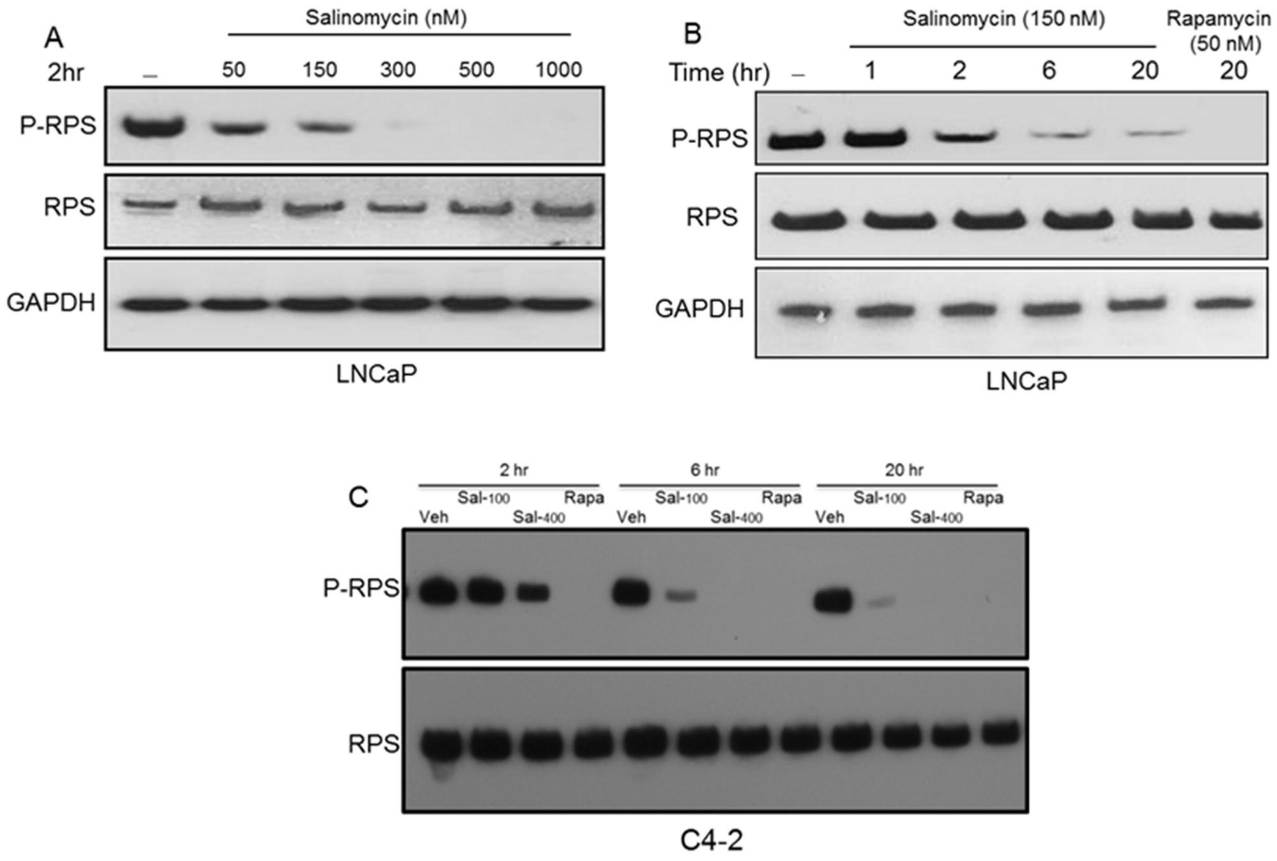

Supplementary Figure S1: Time- and dose-dependent inhibition of RPS phosphorylation by salinomycin. LNCaP **A, B.** C4-2 **C.**

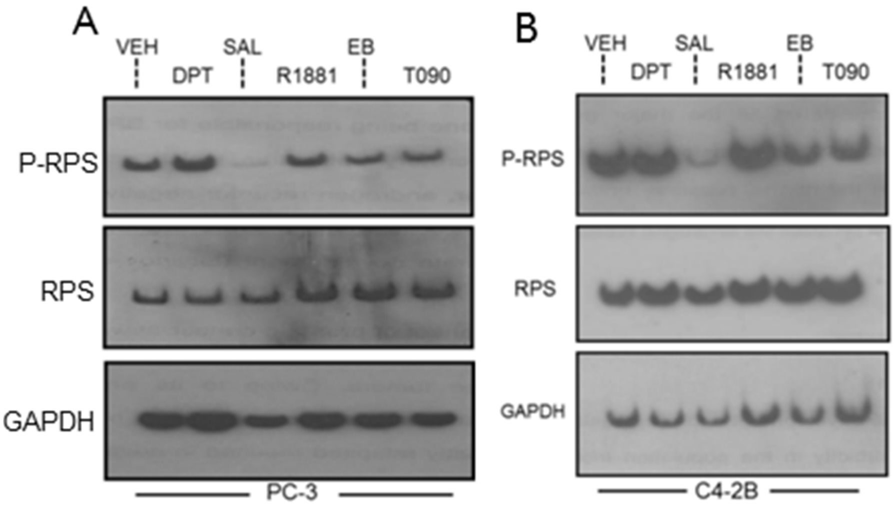

Supplementary Figure S2: P-RPS levels in cells incubated with various prostate cancer cell inhibitors. A. PC3, **B.** C4-2B. DPT: deoxypodophyllotoxin; EB1089(1,25-vitamin D3 analog); T0901317, LXR agonist.

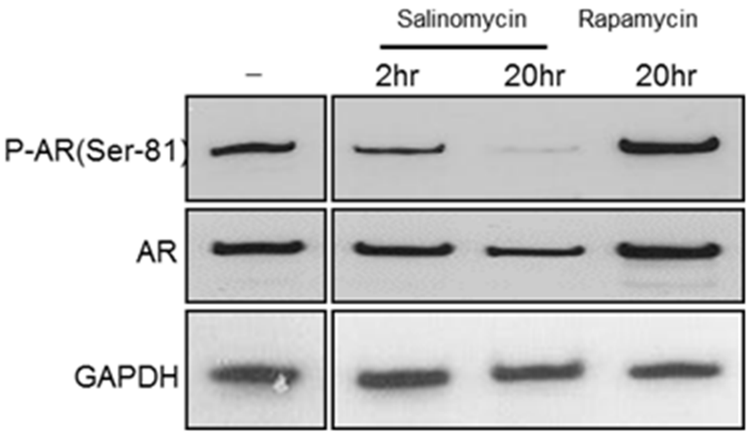

Supplementary Figure S3: Loss of phosphoAR-Ser81 preceded the loss of total AR in salinomycin-incubated cells. C4-2 cells were incubated with 200 nM salinomycin for 20 hours. Unlike salinomycin, rapamycin increased the phosphoAR-Ser81 level.

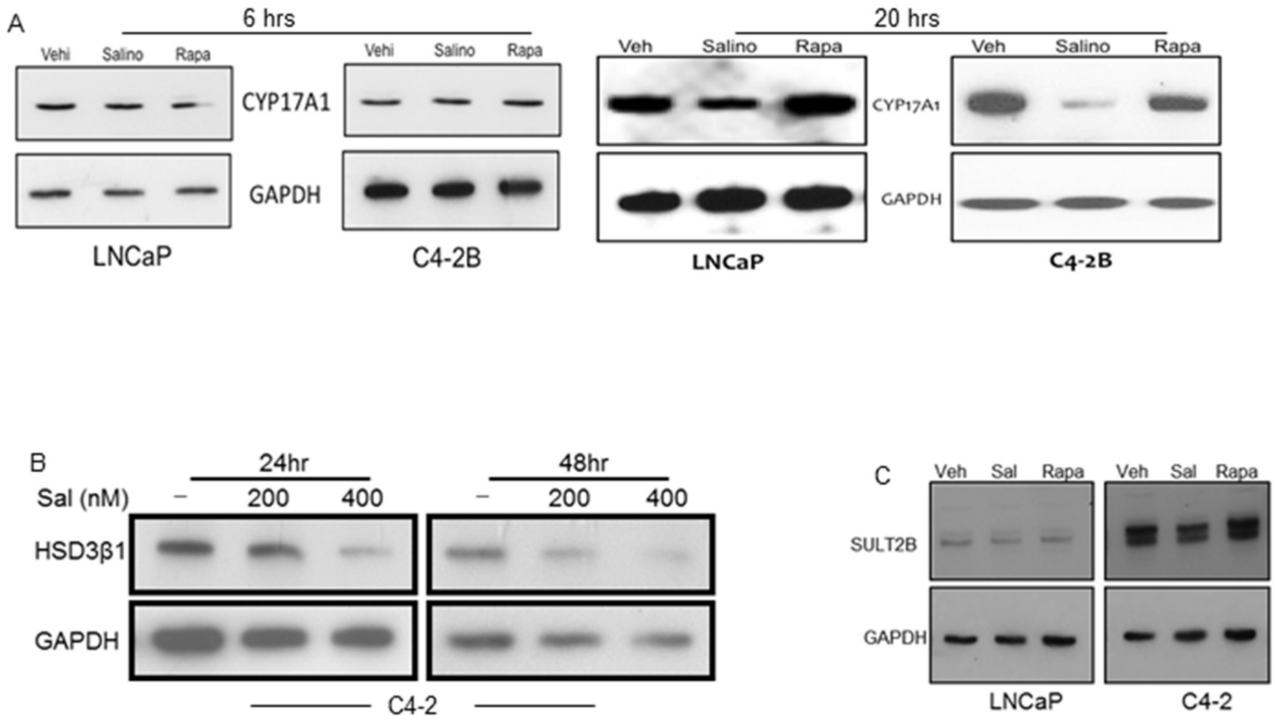

Supplementary Figure S4: CYP17A1, HSD3β1, SULT2B protein levels at two time points in salinomycin-incubated cells. A. CYP17A1 in multiple cell lines at 6hr and 20hr post-treatment with salinomycin (400 nM) and rapamycin (50 nM). **B.** HSD3β1 levels in C4-2 cells incubated with salinomycin (200 nM and 400 nM) for 24 hours and 48 hours. **C.** SULT2B sulfotransferase levels in cells treated for 20 hrs with salinomycin (400 nM) and rapamycin (50 nM).

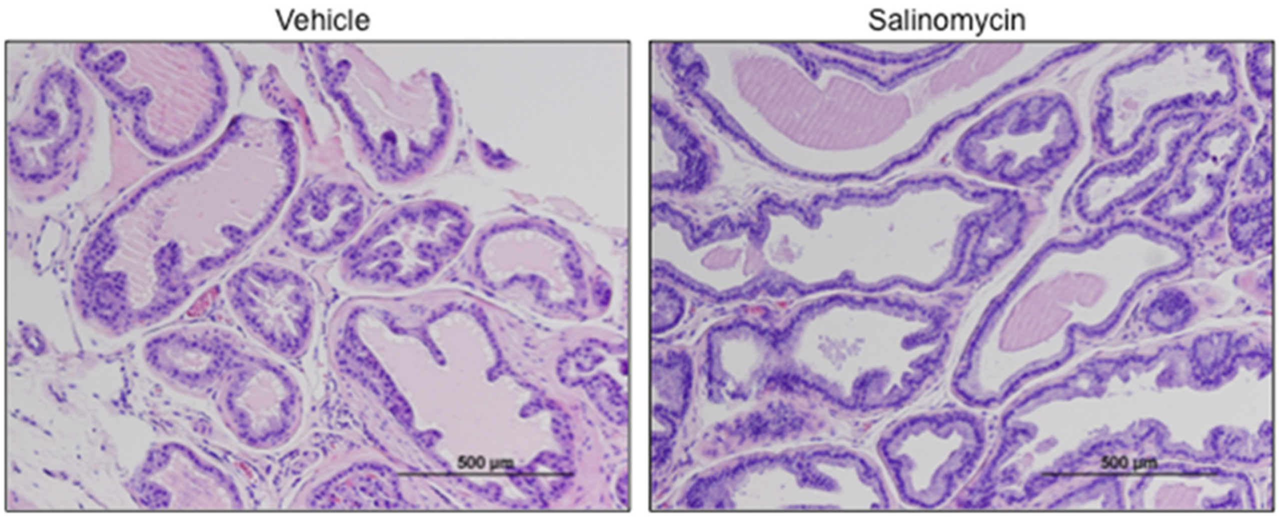

Supplementary Figure S5: Histology of mouse prostate. H&E stained paraffin sections of prostates from vehicle- and salinomycin-injected mice with subcutaneously produced LNCaP-II tumor xenografts.

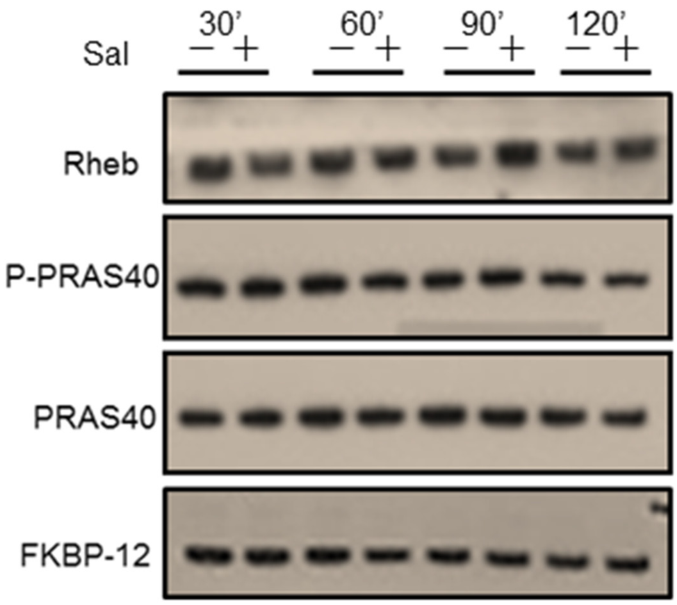

Supplementary Figure S6: Unchanged levels of P-PRAS40, PRAS40, Rheb, FKBP12. C4-2 cells were treated for 20 hours with vehicle or Sal (400 nM).

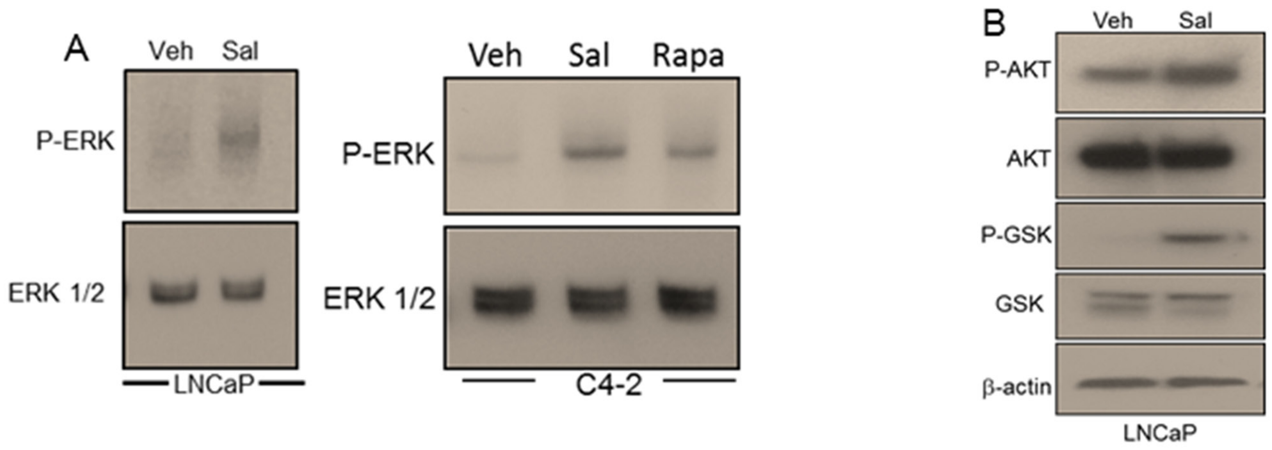

Supplementary Figure S7: Activation of ERK1/2 Map kinase. A. ERK1/2 phosphorylation in LNCaP and C4-2 cells treated for 20 hours with 400 nM salinomycin. **B.** Phosphorylation of AKT-Thr308 and GSK (an AKT substrate) in LNCaP cells.
